# Supplementary material for: The doublecortin-family kinase ZYG-8DCLK1 regulates motor activity to achieve proper force balance in C. elegans acentrosomal spindles
Source: bioRxiv. 2023 Nov 22:2023.11.22.568242. Preprint. [Version 1] doi: 10.1101/2023.11.22.568242 (PMC10690225; doi:10.1101/2023.11.22.568242)
Supplement: Supplement 1 [file NIHPP2023.11.22.568242v1-supplement-1.pdf]

bioRxiv preprint doi: https://doi.org/10.1101/2023.11.22.568242; this version posted November 22, 2023. The copyright holder for this preprint (which was not certified by peer review) is the author/funder, who has granted bioRxiv a license to display the preprint in perpetuity. It is made available under aCC-BY 4.0 International license.

FIGURE S1

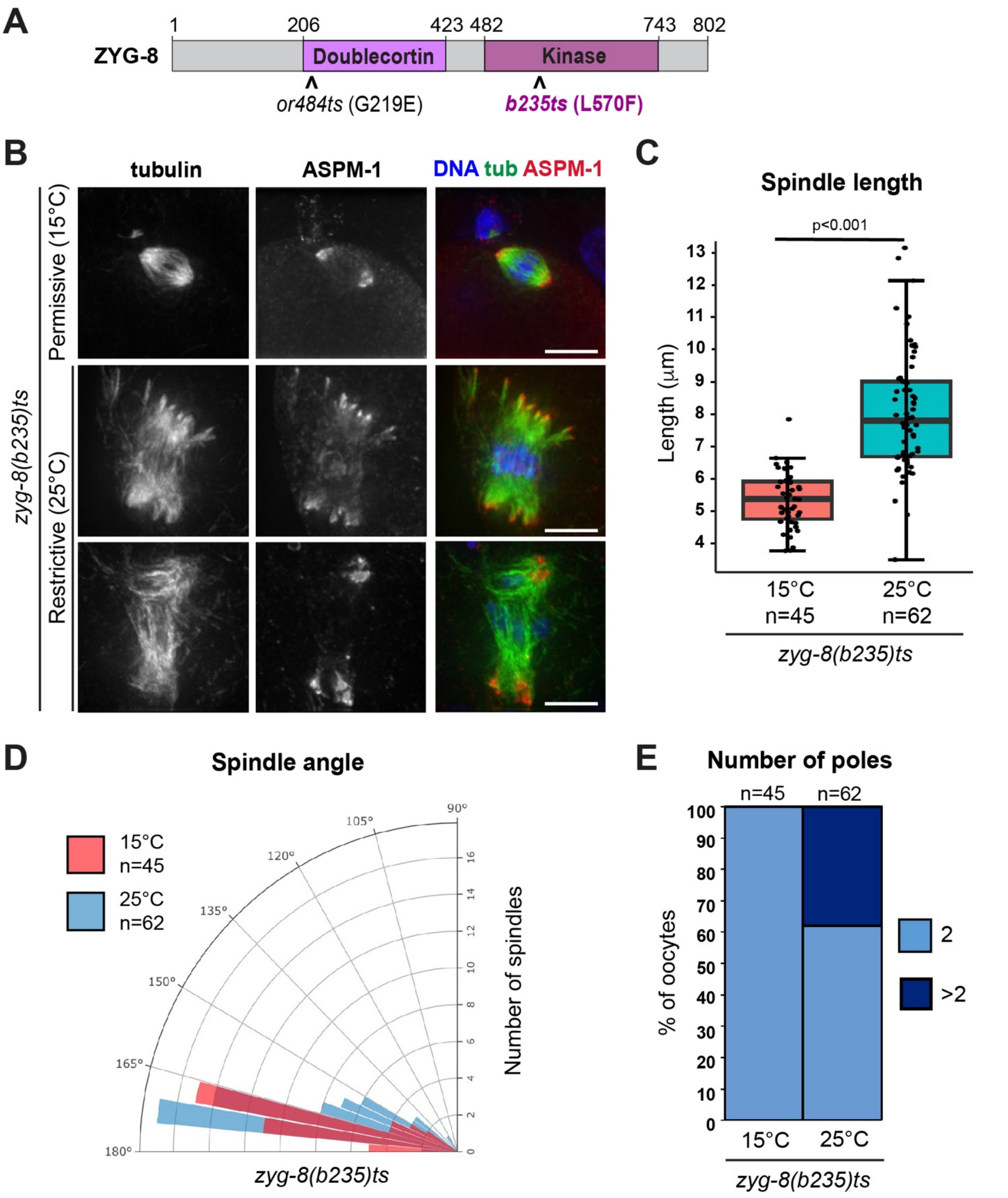

bioRxiv preprint doi: https://doi.org/10.1101/2023.11.22.568242; this version posted November 22, 2023. The copyright holder for this preprint (which was not certified by peer review) is the author/funder, who has granted bioRxiv a license to display the preprint in perpetuity. It is made available under aCC-BY 4.0 International license.

Figure S1. A *zyg-8* temperature sensitive mutant displays defects in oocyte spindle morphology at the restrictive temperature

(A) ZYG-8 schematic, highlighting the microtubule binding doublecortin domain, the kinase domain, and the location of the *b235* temperature sensitive mutation. (B) Immunofluorescence images of *zyg-8(b235)* oocytes at either the permissive (15°C) or restrictive (25°C) temperatures. Shown are tubulin (green), DNA (blue), and ASPM-1 (red). (C-E) Quantification of spindle length, spindle angle, and number of ASPM-1-marked poles in the experiment shown in B. After incubation at the restrictive temperature, oocyte spindles were on average longer, more bent, and some had additional ASPM-1-marked poles. Scale bars = 5µm.

bioRxiv preprint doi: https://doi.org/10.1101/2023.11.22.568242; this version posted November 22, 2023. The copyright holder for this preprint (which was not certified by peer review) is the author/funder, who has granted bioRxiv a license to display the preprint in perpetuity. It is made available under aCC-BY 4.0 International license.

FIGURE S2

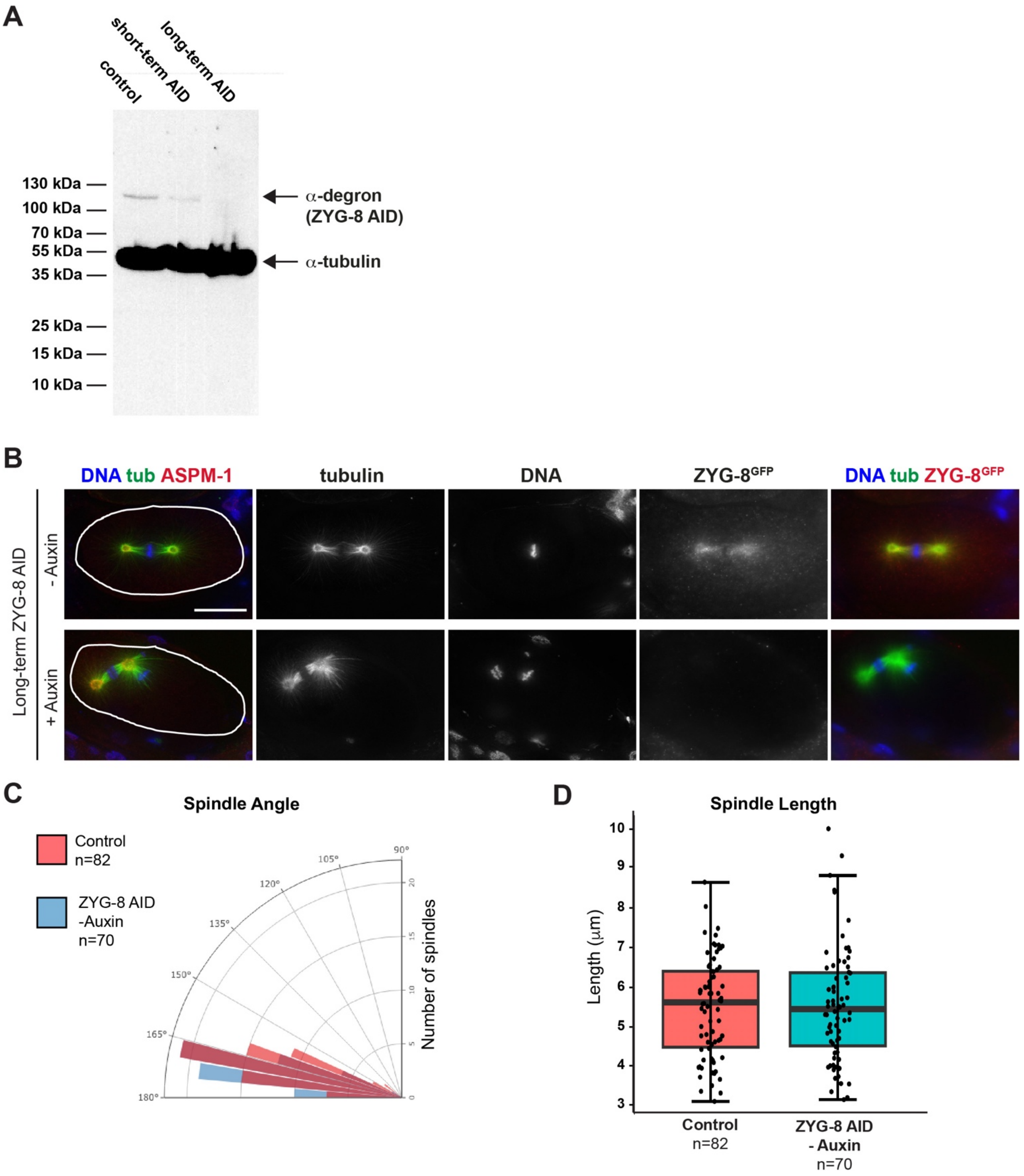

bioRxiv preprint doi: https://doi.org/10.1101/2023.11.22.568242; this version posted November 22, 2023. The copyright holder for this preprint (which was not certified by peer review) is the author/funder, who has granted bioRxiv a license to display the preprint in perpetuity. It is made available under aCC-BY 4.0 International license.

Figure S2. AID allows for temporally and spatially controlled depletion of ZYG-8

(A) Western blot of control, short-term auxin treated (soaking worms for 40 minutes in auxin-containing media), and long-term auxin treated (incubating worms for 18 hours on auxin-containing plates) embryo-only samples. An anti-degron antibody was used to detect ZYG-8 and an anti-tubulin antibody was used as a loading control. (B) IF images of one-cell mitotically dividing embryos in the ZYG-8 AID strain. Auxin treatment resulted in spindle positioning defects (13/15 embryos), phenocopying previous studies of *zyg-8* mutants (Gonczy et al., 2001; Bellanger et al., 2007). (C) Quantification of spindle angle and spindle length in the ZYG-8 AID strain compared to a control strain expressing TIR1 without ZYG-8 tagged; the lengths and angles did not appear significantly different ($p>0.5$), suggesting that tagging ZYG-8 does not substantially alter protein function. Scale bar = 10μm.

bioRxiv preprint doi: https://doi.org/10.1101/2023.11.22.568242; this version posted November 22, 2023. The copyright holder for this preprint (which was not certified by peer review) is the author/funder, who has granted bioRxiv a license to display the preprint in perpetuity. It is made available under aCC-BY 4.0 International license.

FIGURE S3

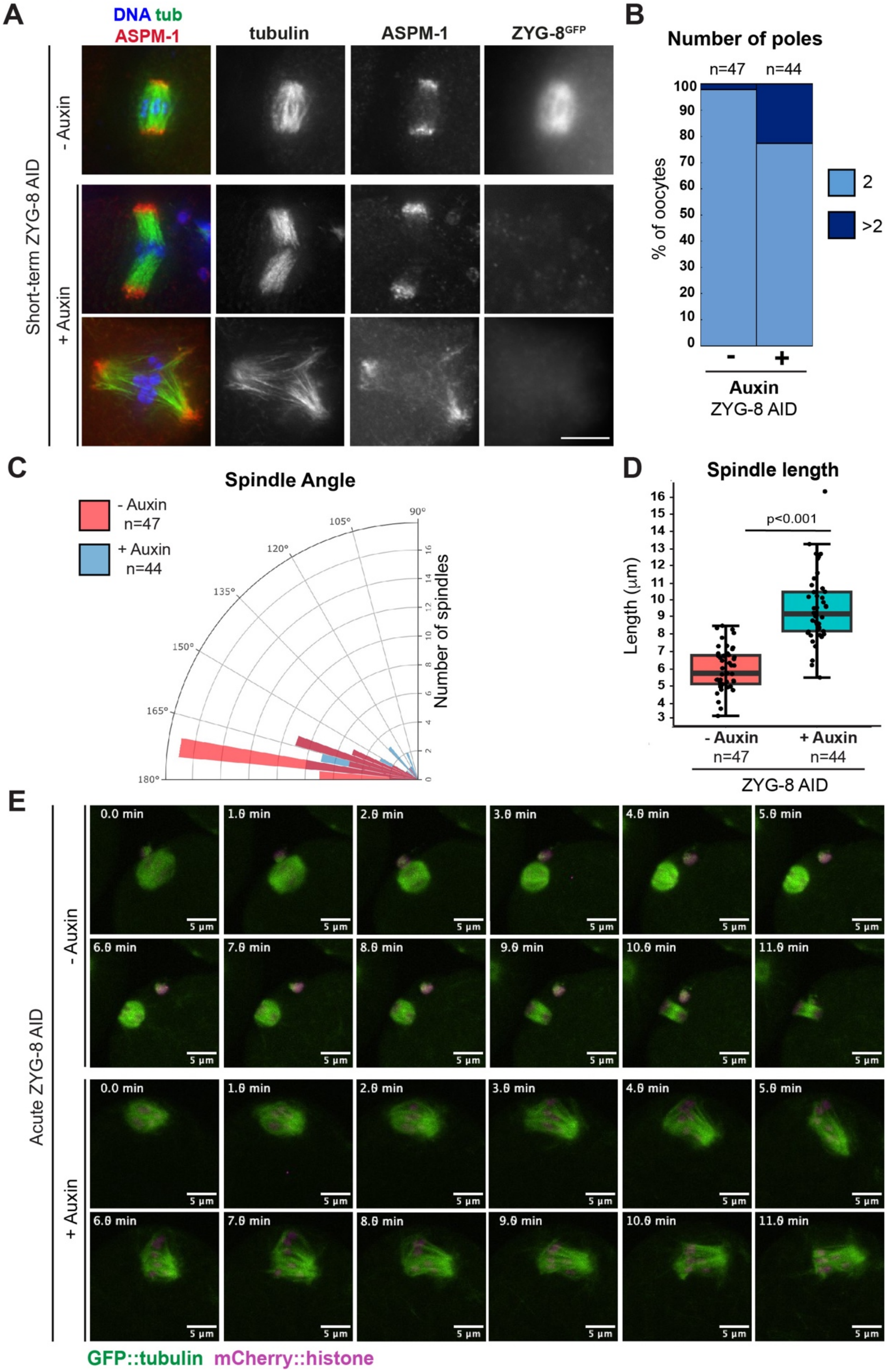

bioRxiv preprint doi: https://doi.org/10.1101/2023.11.22.568242; this version posted November 22, 2023. The copyright holder for this preprint (which was not certified by peer review) is the author/funder, who has granted bioRxiv a license to display the preprint in perpetuity. It is made available under aCC-BY 4.0 International license.

Figure S3. Short-term ZYG-8 depletion in unarrested oocytes reveals the same phenotypes observed following metaphase-arrest

(A) Immunofluorescence images of unarrested oocytes treated with vehicle (row 1) or short-term auxin (rows 2-3); shown are tubulin (green), DNA (blue), and ASPM-1 (red). (B-D) Quantification of the number of ASPM-1-marked poles, spindle angle, and spindle length. Short term ZYG-8 AID results in spindle defects even without metaphase arrest. (E) Live imaging of acute auxin treatment of unarrested spindles; shown are GFP::tubulin (green) and mCherry::histone (magenta). Control spindles maintain bipolarity and eventually segregate chromosomes in anaphase (rows 1-2). In contrast, rows 3-4 show an auxin-treated unarrested spindle elongate and weaken at the midspindle, demonstrating the same defects observed with metaphase arrest. Scale bars = 5μm.

bioRxiv preprint doi: https://doi.org/10.1101/2023.11.22.568242; this version posted November 22, 2023. The copyright holder for this preprint (which was not certified by peer review) is the author/funder, who has granted bioRxiv a license to display the preprint in perpetuity. It is made available under aCC-BY 4.0 International license.

FIGURE S4

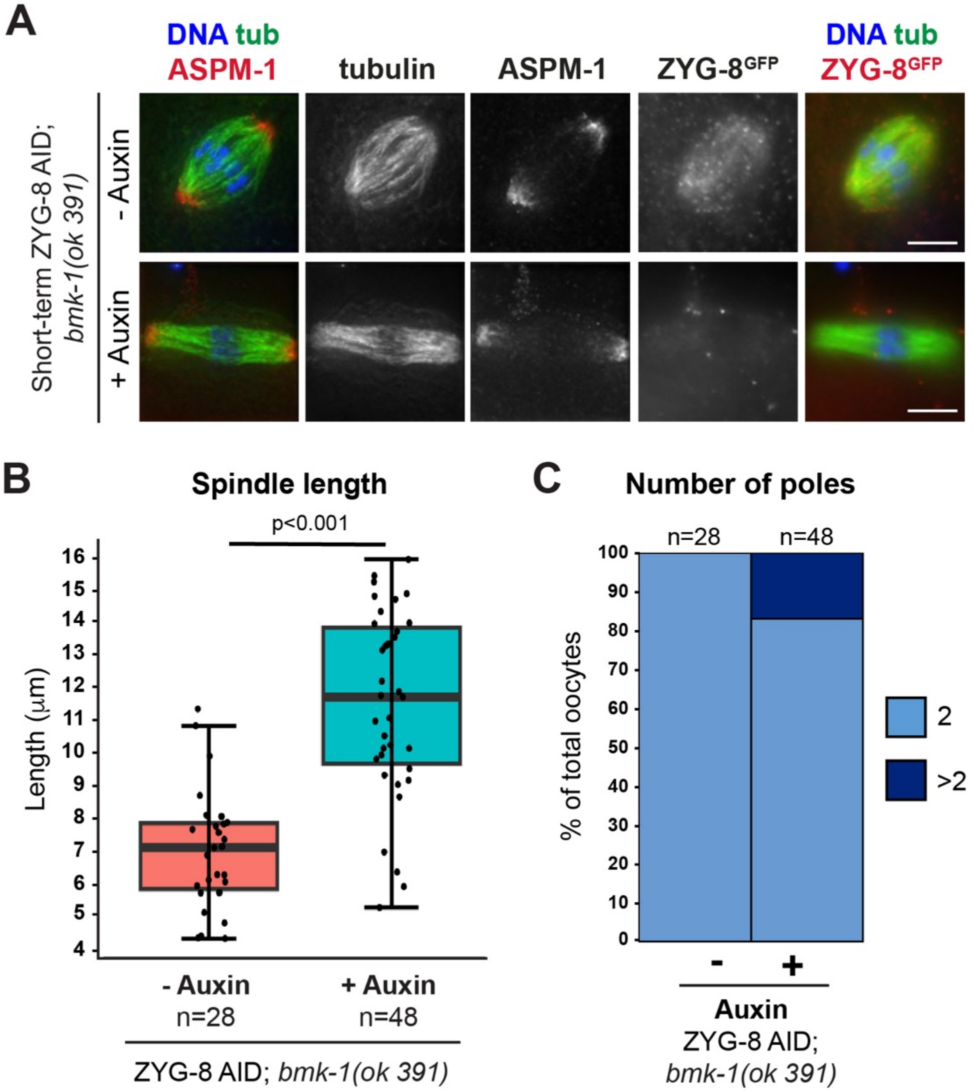

652 **Figure S4. ZYG-8 depletion causes spindle phenotypes in a *bmk-1* mutant without KLP-18 depletion**

(A) Immunofluorescence images of oocyte spindles in the ZYG-8 AID; *bmk-1(ok391)* strain in the presence and absence of auxin. Due to diffuse ZYG-8 localization, ZYG-8 images are not deconvolved. (B-C) Quantification of spindle length and the number of ASPM-1-marked poles per spindle. Spindles are longer upon auxin depletion and a fraction of spindles have multiple poles, demonstrating that ZYG-8 likely has functions in addition to regulating BMK-1. Scale bars = 5μm.

bioRxiv preprint doi: https://doi.org/10.1101/2023.11.22.568242; this version posted November 22, 2023. The copyright holder for this preprint (which was not certified by peer review) is the author/funder, who has granted bioRxiv a license to display the preprint in perpetuity. It is made available under aCC-BY 4.0 International license.

VIDEO LEGENDS

Video 1. ZYG-8 AID metaphase-arrested oocyte spindles maintain bipolarity in the absence of auxin

Live imaging of an *emb-30(RNAi)* metaphase-arrested oocyte spindle; corresponds to Figure 3A. Shown are GFP::tubulin (green) and mCherry::histone (magenta). Oocytes were dissected into control Meiosis Medium containing vehicle. The spindle maintains bipolarity; chromosomes oscillate but stay aligned at the midspindle. The phenotype was consistent in all videos (n=5). Scale bar = 5μm.

Video 2. ZYG-8 AID metaphase-arrested oocyte spindles elongate and lose mid-spindle integrity upon auxin treatment

Live imaging of an *emb-30(RNAi)* metaphase-arrested oocyte spindle; corresponds to Figure 3A. Shown are GFP::tubulin (green) and mCherry::histone (magenta). Oocytes were dissected into auxin-containing Meiosis Medium. The spindle immediately begins to elongate, the midspindle weakens, and chromosomes lose alignment at the midspindle. The phenotype was consistent in all videos (n=10). Scale bar = 5μm.

Video 3. ZYG-8 AID unarrested oocyte spindles maintain bipolarity and undergo anaphase in the absence of auxin

Live imaging of a control oocyte spindle; corresponds to Figure S3E. Shown are GFP::tubulin (green) and mCherry::histone (magenta). Oocytes were dissected into control Meiosis Medium containing vehicle. The spindle maintains bipolarity in metaphase, rotates towards the cortex, shortens, and then elongates as chromosomes segregate bidirectionally. The phenotype was consistent in all videos (n=5). Scale bar = 5μm.

bioRxiv preprint doi: https://doi.org/10.1101/2023.11.22.568242; this version posted November 22, 2023. The copyright holder for this preprint (which was not certified by peer review) is the author/funder, who has granted bioRxiv a license to display the preprint in perpetuity. It is made available under aCC-BY 4.0 International license.

Video 4. ZYG-8 AID unarrested oocytes exhibit spindle defects following auxin treatment

Live imaging of an unarrested auxin-treated oocyte spindle; corresponds to Figure S3E. Shown are GFP::tubulin (green) and mCherry::histone (magenta). Oocytes were dissected into auxin-containing Meiosis Medium. The auxin-treated spindle begins to elongate as the midspindle loses integrity, and chromosome become misaligned. Spindle defects were observed in all videos (n=5). Scale bar = 5μm.

Video 5. *klp-18(RNAi)* spindles maintain a single monopole as chromosomes move towards the center of the aster during anaphase

Live imaging of a *klp-18(RNAi)* ZYG-8 AID oocyte spindle; corresponds to Figure 5C. Shown are GFP::tubulin (green) and mCherry::histone (magenta). Oocytes were dissected into control Meiosis Medium containing vehicle. Control *klp-18(RNAi)* spindles remain monopolar as chromosomes slowly move towards the center pole in anaphase. The phenotype was consistent in all videos (n=5). Scale bar = 5μm.

Video 6. Acute ZYG-8 AID causes monopolar spindles to reorganize, reestablish bipolarity, and segregate chromosomes bidirectionally, example 1

Live imaging of a *klp-18(RNAi)* ZYG-8 AID oocyte spindle; corresponds to Figure 5C. Shown are GFP::tubulin (green) and mCherry::histone (magenta). Oocytes were dissected into auxin-containing Meiosis Medium. Upon treatment with auxin, the monopolar spindle reorganizes into a bipolar spindle that then segregates chromosomes bidirectionally (bidirectional chromosome segregation was observed in 4/12 oocyte spindles). Scale bar = 5μm.

Video 7. Acute ZYG-8 AID causes monopolar spindles to reorganize and reestablish bipolarity, example 2

Live imaging of a *klp-18(RNAi)* ZYG-8 AID Meiosis II oocyte spindle; corresponds to Figure 5C. Shown are GFP::tubulin (green) and mCherry::histone (magenta). Oocytes were dissected into

bioRxiv preprint doi: https://doi.org/10.1101/2023.11.22.568242; this version posted November 22, 2023. The copyright holder for this preprint (which was not certified by peer review) is the author/funder, who has granted bioRxiv a license to display the preprint in perpetuity. It is made available under aCC-BY 4.0 International license.

auxin-containing Meiosis Medium. Upon treatment with auxin, the monopolar spindle reorganizes and incorporates the polar body, forming a multipolar and finally a bipolar spindle. Monopolar spindles reorganized in 12/12 videos: 3/12 reincorporated the polar body, 6/12 reestablished bipolarity, and 3/12 formed disorganized spindles. Scale bar = 5μm.

Video 8. ZYG-8 AID; *bmk-1(ok391)* monopolar spindles maintain a single pole and chromosomes move inwards in anaphase in the absence of auxin

Live imaging of a *klp-18(RNAi)* ZYG-8 AID *bmk-1(ok391)* oocyte spindle; corresponds to Figure 6C. Shown are GFP::tubulin (green) and mCherry::histone (magenta). Oocytes were dissected into control Meiosis Medium containing vehicle. The monopolar spindle maintains a single pole as chromosomes move towards the center of the spindle in anaphase. The phenotype was consistent in all videos (n=5). Scale bar = 5μm.

Video 9. ZYG-8 AID; *bmk-1(ok391)* monopolar spindles maintain a single pole and chromosomes move inwards in anaphase following auxin treatment

Live imaging of a *klp-18(RNAi)* ZYG-8 AID *bmk-1(ok391)* oocyte spindle; corresponds to Figure 6C. Shown are GFP::tubulin (green) and mCherry::histone (magenta). Oocytes were dissected into auxin-containing Meiosis Medium. After auxin treatment, the monopolar spindle maintains a single pole as chromosomes move towards the center of the spindle in anaphase. The phenotype was consistent in all videos (n=5). Scale bar = 5μm.
